# Beta receptor-mediated modulation of the oddball P3 but not error-related ERP components in humans

**DOI:** 10.1007/s00213-015-3966-2

**Published:** 2015-07-04

**Authors:** Mischa de Rover, Stephen B. R. E. Brown, Guido P. Band, Erik J. Giltay, Martijn S. van Noorden, Nic J. A. van der Wee, Sander Nieuwenhuis

**Affiliations:** Clinical Psychology Unit, Institute of Psychology, Leiden University, Wassenaarseweg 52, 2333 AK Leiden, The Netherlands; Leiden Institute for Brain and Cognition (LIBC), Leiden University, Leiden, The Netherlands; Cognitive Psychology Unit, Institute of Psychology, Leiden University, Leiden, The Netherlands; Department of Psychiatry, Leiden University Medical Center, Leiden, The Netherlands

**Keywords:** Beta-blockers, Norepinephrine, P300, ERP, EEG, Error processing, Post-error slowing, Conflict adaptation, Antagonist, Inverted U

## Abstract

**Rationale:**

The P3 is a ubiquitous component of stimulus-driven neural activity that can be observed in scalp electrophysiological recordings. Multiple lines of evidence suggest an important role for the noradrenergic system in the generation of the P3. However, pharmacological studies of the P3 using noradrenergic manipulations have so far been limited to agents that affect α2-receptor signaling.

**Objectives:**

The present study investigated whether β-adrenergic receptors are involved in the generation of the P3 and the error positivity (Pe), a component of the event-related potential that is elicited by errors and that bears many similarities to the P3.

**Methods:**

We used a double-blind, placebo-controlled, crossover design in which we examined in human participants (*N* = 16) the effect of a single dose of propranolol (80 mg) on the amplitudes of the P3 observed in visual and auditory oddball tasks and the Pe observed in a flanker task.

**Results:**

We found that P3s to auditory stimuli were increased in amplitude following treatment with propranolol. Propranolol also modulated the P3 to visual stimuli, but in a direction dependent on participants’ level of trait anxiety: In participants with lower trait anxiety, propranolol resulted in a (non-significant) decrease in P3 amplitudes; in participants with higher trait anxiety, propranolol significantly enhanced P3 amplitude. Propranolol did not modulate the amplitude of the Pe or behavioral measures of conflict/error-related performance adjustments.

**Conclusions:**

These results provide the first evidence for involvement of β-adrenergic receptors in P3 generation. We speculate that propranolol affected the P3 through actions at β2-receptors in the locus coeruleus.

## Introduction

The P3 (or P300) is a ubiquitous component of stimulus-driven neural activity that can be observed in electroencephalography (EEG) recordings. Researchers generally agree that the P3 must reflect key aspects of information processing, such as updating of memory (Donchin and Coles 1988; see also Nieuwenhuis 2011), decision making (O’Connell et al. 2012; Verleger et al. 2005), and temporal filtering (Nieuwenhuis et al. 2005). To understand the neurochemical mechanisms underlying generation of the P3, a large number of studies have examined the effects on the P3 of pharmacological manipulations that affect one or several neurotransmitter or neuromodulator systems. These studies have implicated a variety of neurochemical systems in P3 generation (Frodl-Bauch et al. 1999; Polich 2007; Soltani and Knight 2000), but the most consistent P3 effects have probably been obtained in pharmacological studies of the noradrenergic system. This system consists of the brainstem nucleus locus coeruleus (LC) and widespread ascending projections throughout the brain, where LC activity leads to the release of the neuromodulator norepinephrine (noradrenaline) (Berridge and Waterhouse 2003). There is a wealth of evidence that the P3 reflects, at least in part, the neuromodulatory effect of phasic norepinephrine release in the neocortex (de Taeye et al. 2014; Nieuwenhuis 2011; Nieuwenhuis et al. 2005). The primary goal of the current study was to examine in more detail the role of the noradrenergic system in generation of the P3. The second goal was to test for noradrenergic modulation of the error positivity (Pe), a broad positive event-related potential (ERP) component elicited by errors that shows many similarities to the P3.

Noradrenergic signaling occurs through three major categories of receptors: α1, α2, and β, each type being associated with different cellular responses (Berridge and Waterhouse 2003) and cognitive functions (Chamberlain and Robbins 2013). Therefore, it is surprising that P3 studies using noradrenergic manipulations have been limited to agents that affect α2-receptor signaling—mostly clonidine, an α2-receptor agonist that at moderate doses decreases noradrenergic activity. Although the results are not unequivocal (e.g., Shelley et al. 1997), the large majority of these studies has found that clonidine decreases P3 amplitude, especially in auditory target detection tasks (Logemann et al. 2013; Turetsky and Fein 2002; other studies are reviewed in Nieuwenhuis et al. 2005).

So far, there have been no published studies that examined the role of β-receptor signaling in P3 generation. This is surprising given the strong relationship between P3 amplitude and learning (Donchin and Coles 1988; see also Nieuwenhuis 2011) and the fact that norepinephrine promotes long-term potentiation, a prominent neural substrate for learning and memory, through actions at β-adrenergic receptors (Gibbs and Summers 2002). Strange and Dolan (2007) found that propranolol, a centrally acting β-receptor antagonist, abolished the large blood–oxygen level-dependent (BOLD) response elicited by oddball targets in areas that receive strong noradrenergic projections and that contribute significantly to the scalp-recorded P3. This suggests that propranolol should decrease oddball P3 amplitude. Testing this hypothesis was the primary aim of our study.

Several authors have noted the similarities between the P3 and the Pe (Leuthold and Sommer 1999; Overbeek et al. 2005; but see Falkenstein et al. 2000), a positive component that typically peaks between 200 and 400 ms after incorrect responses, immediately following the error-related negativity (ERN). The P3 and Pe have a similar polarity, morphology, and timing, and both components are elicited by motivationally significant events, which has led to the proposal that the Pe may constitute a P3 associated with the motivational significance of an error (Overbeek et al. 2005; Rösler 1983). Recent principal component analyses of the Pe have shown that it consists of two subcomponents, a fronto-central component with a similar spatial distribution as the error-related negativity and a centro-parietal component closely resembling the P3 (Arbel and Donchin 2009; Endrass et al. 2012). Intracranial recordings in humans suggest that the Pe, like the P3 (Nieuwenhuis et al. 2005), has widely distributed cortical sources (Brázdil et al. 2002; see also Helenius et al. 2010). Furthermore, two studies have reported a significant positive across-subject correlation between the amplitude of the Pe and the amplitude of the P3 recorded in the same task (Davies et al. 2001) or a different task (Ridderinkhof et al. 2009). Finally, the two components have both been argued to reflect a decision variable that integrates noisy evidence until it reaches a boundary-crossing criterion: While the P3 may reflect the accumulation of evidence that a target stimulus has occurred (Nieuwenhuis 2011; O’Connell et al. 2012), the Pe may reflect accumulated evidence that an error has been committed (Murphy et al. 2012; Steinhauser and Yeung 2010).

These results, indicating many parallels between the P3 and Pe, suggest that pharmacological agents that affect the P3 may also affect the Pe. The secondary aim of our study was to test this prediction. We examined the effects of a single dose of propranolol (80 mg) on P3 and Pe amplitude using a double-blind, placebo-controlled crossover design. P3s were elicited using visual and auditory oddball tasks; Pes were elicited by errors in a flanker task. Because of the inverted U-shaped relationship between baseline noradrenergic activity and phasic noradrenergic responses (Aston-Jones and Cohen 2005), the effects of noradrenergic drugs on behavioral and neural correlates of phasic noradrenergic responses (including the P3) may critically depend on an individual’s natural baseline level of noradrenergic activity (e.g., Coull 1994; Luksys et al. 2009). Therefore, we collected a measure of trait anxiety, which strongly correlates with baseline noradrenergic activity (Howells et al. 2012; Ressler and Nemeroff 2000), and examined if the level of trait anxiety interacted with the effect of treatment.

## Methods

### Participants

Sixteen healthy young adults (10 women), aged 18–28 years (average age 22.0 years ± 3.2 standard deviation (SD)), were included in the study in return for 100€. Only participants with a systolic blood pressure above 100 mmHg (average 126.6 ± 15.6 SD), a diastolic blood pressure above 60 mmHg (average 69.3 ± 7.1 SD), and a heart rate above 60 beats per minute (average 75.4 ± 10.9 SD) were included in the study. All participants underwent a medical screening and were considered to be in satisfactory health. The use of medication that could interfere with propranolol was stopped the day before. Participants received an oral dose of 80 mg propranolol or placebo in a randomized, double-blind, counterbalanced crossover design. Propranolol and placebo were administered to each participant on consecutive days (24 h in between administrations) with both sessions at the same time of day. EEG was recorded in both sessions during the performance of the oddball and flanker task (described in detail below). The data from one additional participant could not be collected because of severe side effects of propranolol (de Rover et al. 2010), and the data from one other additional participant was excluded because he did not complete the tasks. The data from 16 participants were analyzed. The study was approved by the medical ethics committee of the Leiden University Medical Center and was conducted according to the Declaration of Helsinki. Informed consent was obtained from all participants before their inclusion in the study.

### Procedure

**Fig. 1:**
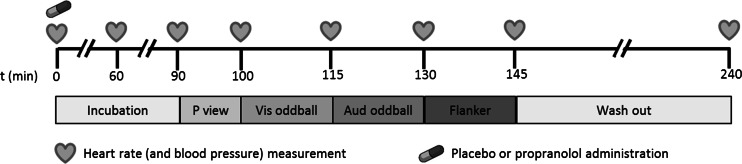
Timeline of the experiment. *P view* passive viewing task (de Rover et al. 2012), *Vis oddball* visual oddball task, *Aud oddball* auditory oddball task, *Flanker* Flanker task, *Wash out* wash out period, to allow the heart rate to return to (approximately) baseline

Participants were instructed to abstain from caffeine, nicotine, alcohol, and other psychoactive substances from 15 h before the start of the first session until the end of the second session (the next day). After checking if blood pressures and heart rate were still meeting the inclusion criteria, participants received a microcrystalline cellulose-filled capsule with either propranolol or placebo (*t* = 0). Propranolol has well-established antihypertensive properties; therefore, blood pressure and heart rate were monitored for participants’ safety. Measurements were taken at *t* = 0, *t* = 60, *t* = 90, *t* = 100, *t* = 115, *t* = 130, *t* = 145, and *t* = 240. Between *t* = 100 and *t* = 145, participants performed the three tasks reported here in fixed order (Fig. 1). Given the slow pharmacokinetics of orally administered propranolol (*T*_max_ = 1–2 h, *T*_1/2_ = 3–6 h), it is unlikely that any differences in results between tasks were due to this fixed order. After completion of the tasks, the participants were debriefed and paid. At *t* = 240, participants were reevaluated and sent home if blood pressure and heart rate were (near) normal.

### Tasks and questionnaire

Participants first performed a visual and an auditory oddball task, in fixed order. On each trial of the visual oddball task, a black cross or circle (1.1 × 1.1°) was presented for 250 ms on a light grey background. Participants were instructed to make speeded key press responses with the dominant hand to target stimuli (circles, 20 % of the trials) but not to non-target stimuli (crosses, 80 % of the trials). On each trial of the auditory oddball task, a 150-ms tone (75 dB) was presented. Participants were instructed to fixate on a centrally presented fixation cross and to make speeded key press responses with the dominant hand to target tones (2000-Hz tones, 20 % of the trials) but not to non-target tones (1000-Hz tones, 80 % of the trials). In both tasks, the time interval between the onsets of two successive stimuli was 2.5 s. Each task consisted of 150 experimental trials: 30 target trials and 120 non-target trials. Two participants were excluded from the analysis of the auditory oddball task data because of technical problems with the EEG recording.

Next, participants performed a flanker task. Stimuli were presented in white against a dark grey background on a computer screen placed at a distance of 92 cm from the participant. Each stimulus array subtended a visual angle of 5.5 × 0.6° and consisted of five horizontally arranged letters displayed in Arial font: HHHHH, SSSSS, HHSHH, or SSHSS. The participants were instructed to respond “as quickly and accurately as possible” to the central target letter and to try to ignore the four flankers. The H was mapped to the left and the S to the right response key. A distinction was made between congruent (i.e., target letter and flankers associated with the same response; e.g., HHHHH) and incongruent (i.e., target letter and flankers associated with different responses; e.g., HHSHH) stimulus arrays. Each trial started with a 500-ms fixation cross, followed by the presentation of the stimulus array for 100 ms and a blank screen until the response was registered. The response was followed by another 500-ms blank screen, after which the next trial started. Each participant completed six blocks of 80 trials each. Each block contained 20 trials with each of the four possible stimulus arrays. Presentation order of the stimulus arrays was randomized. After each block, the participants received verbal feedback encouraging them to speed up or slow down their responding, with the aim of maintaining accuracy between 80 and 85 % correct. One participant was excluded from the analysis of the flanker task data because of technical problems with the EEG recording.

In a separate session, the participants filled out the 20-item trait scale of the State-Trait Anxiety Inventory for Adults (STAI; Spielberger et al. 1983), a standard anxiety questionnaire that measures trait anxiety on a 4-point scale.

### EEG recording and analyses

We recorded EEG from 31 Ag/AgCl scalp electrodes (Fp1, AFz, Fz, F3, F7, FCz, FC3, FT7, Cz, C3, T7, CPz, CP3, TP7, Pz, P3, P7, POz, O1, O2, P8, P4, TP8, CP4, T8, C4, FT8, FC4, F8, F4, Fp2) and from the left and right mastoids. We measured the horizontal and vertical electro-oculogram (EOG) using bipolar recordings from electrodes placed approximately 1 cm lateral of the outer canthi of the two eyes and from electrodes placed approximately 1 cm above and below the participant’s right eye.

The EEG signal was pre-amplified at the electrode to improve the signal-to-noise ratio and amplified with a gain of 16× by a BioSemi ActiveTwo system (BioSemi B.V., Amsterdam). The data were digitized at 24-bit resolution with a sampling rate of 512 Hz using a low-pass fifth-order sinc filter with a half-power cutoff of 102.4 Hz. Each active electrode was measured online with respect to a common mode sense (CMS) active electrode producing a monopolar (non-differential) channel and was referenced offline to the average of the left and right mastoids. EEG and EOG were high-pass filtered at 0.01 Hz. Ocular and eye blink artifacts were corrected using the method of Gratton et al. (1983). Epochs with other artifacts (spike artifacts (50 μV/2 ms) and slow drifts (200 μV/200 ms)) were also discarded. We extracted single-trial epochs for a period from 200 ms before to 600 ms after stimulus onset. Then, for each participant and stimulus type, we averaged the EEG epochs to create stimulus-locked (oddball tasks) and response-locked (flanker task) event-related potentials (ERPs). The average signal during the 200 ms pre-stimulus or pre-response baseline was subtracted from each ERP. The P3 was defined as the peak amplitude of the signal at electrode POz, where treatment effects on P3 amplitude were most pronounced, in the time window 150–550 ms. However, similar results were obtained if the P3 was defined as the mean amplitude in the time window 230–400 ms. The Pe was defined as the peak amplitude of the signal at electrode FCz in the time window 100–500 ms (Overbeek et al. 2005). The ERN was defined as the mean amplitude of the signal at electrode FCz in the time window 0–150 ms. To examine the interaction between treatment and trait anxiety, we included STAI trait score (high or low, according to median split) as an additional between-subject variable in all ERP analyses. The average STAI trait (sum) score over all participants was 33.4 ± 8.6 (SD) with a Cronbach’s alpha of 0.926. For the high-anxiety group, the average STAI trait score was 40.1 ± 6.8 (SD), and for the low-anxiety group, the average STAI trait score was 26.8 ± 3.2 (SD).

## Results

### Cardiovascular measurements

**Fig. 2:**
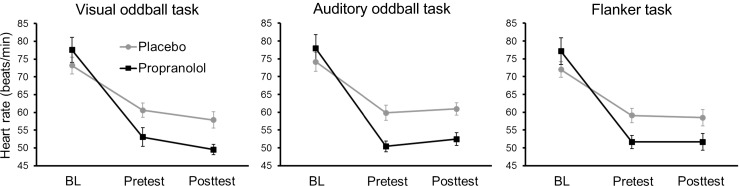
Cardiovascular measurements for each of the three tasks. *BL* baseline (*t* = 0), *Pretest* right before the start of each task, *Posttest* immediately after the end of each task. *Error bars* indicate standard errors of the means

Heart rate was used as a marker to check for successful β-receptor blockade by propranolol (Fig. 2). Heart rate was registered at baseline (*t* = 0), just before the start of each task (pre-test), and immediately after the end of each task (post-test). For each of the three tasks, there was a significant decrease in heart rate during the experiment (visual oddball task: *F*(2,24) = 67.4, *p* < 0.001; auditory oddball task: *F*(2,18) = 50.5, *p* < 0.001; flanker task: *F*(2,24) = 135.2, *p* < 0.001), which was significantly larger for propranolol than for placebo treatment (interaction time × treatment: visual oddball task: *F*(2,24) = 19.7, *p* < 0.001; auditory oddball task: *F*(2,18) = 19.6, *p* < 0.001; flanker task: *F*(2,24) = 21.6, *p* < 0.001). Subsequent pairwise comparisons showed that baseline heart rate did not differ between propranolol and placebo treatment (visual oddball task: *t*_15_ = 1.7, *p* = 0.11; auditory oddball task: *t*_13_ = 1.3, *p* = 0.21; flanker task: *t*_14_ = 2.0, *p* = 0.07), whereas heart rate was significantly lower in the propranolol condition than the placebo condition at pre-test (visual oddball task: *t*_14_ = 2.9, *p* = 0.013; auditory oddball task: *t*_10_ = 4.5, *p* = 0.001; flanker task: *t*_12_ = 4.2, *p* = 0.001) and at post-test measurements (visual oddball task: *t*_12_ = 4.3, *p* = 0.001; auditory oddball task: *t*_11_ = 4.6, *p* = 0.001; flanker task: *t*_14_ = 3.0, *p* = 0.009).

### ERP oddball tasks

**Fig. 3:**
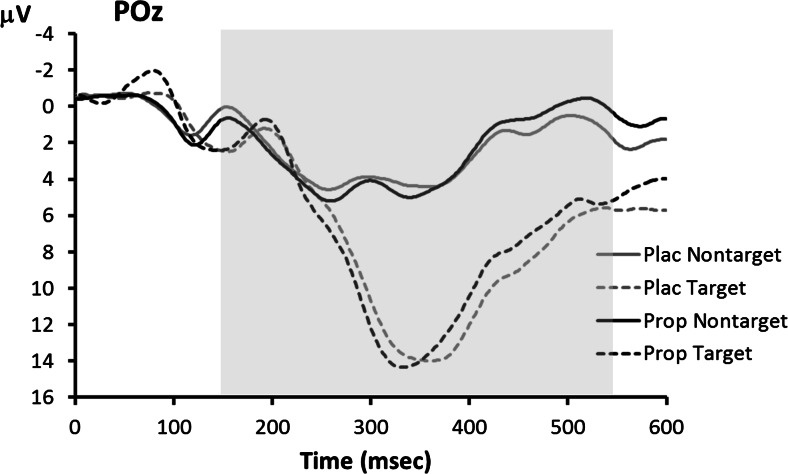
Visual oddball task: grand average ERP waveforms associated with targets and non-targets in the placebo (*Plac*) and propranolol (*Prop*) condition. The *shaded area* indicates the time window used for P3 peak detection

**Fig. 4:**
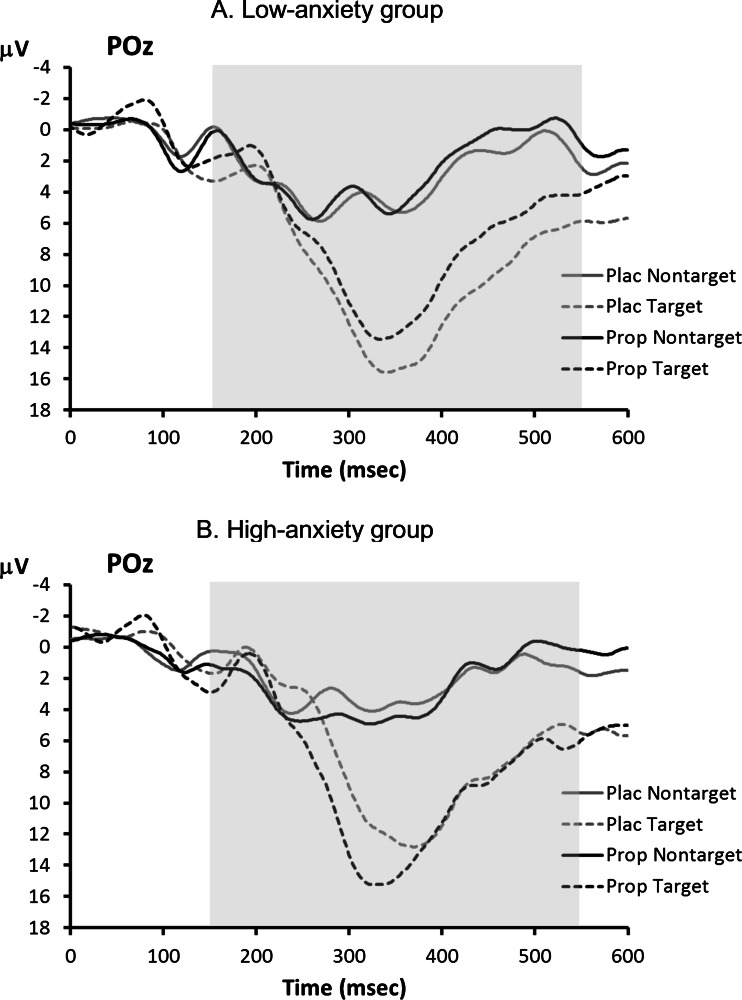
Visual oddball task: average ERP waveforms for **a** low-anxiety participants and **b** high-anxiety participants. The *shaded area* indicates the time window used for P3 peak detection

ERP waveforms for the visual oddball task are shown in Fig. 3. The P3 showed the typical main effect of trial type, indicating larger P3s to target stimuli than to non-target stimuli, *F*(1,14) = 95.5, *p* < 0.001, but no main effect of treatment, *F*(1,14) = 0.3, *p* = 0.58. However, as Fig. 4 shows, the main effect of treatment on P3 amplitude was obscured by a crossover interaction with trait anxiety level, *F*(1,14) = 7.2, *p* = 0.018. We investigated this interaction further using separate ANOVAs for the two anxiety groups. In the low-anxiety group (Fig. 4a), there was no reliable main effect of treatment, *F*(1,7) = 1.5, *p* = 0.26, but note that propranolol treatment led to numerically smaller P3s, especially to targets (placebo 16.5 μV, propranolol 14.4 μV). In contrast, in the high-anxiety group (Fig. 4b), propranolol treatment led to significantly larger P3s, as indicated by a main effect of treatment: *F*(1,7) = 10.0, *p* = 0.016 (target P3s—placebo 9.8 μV, propranolol 11.7 μV).

**Fig. 5:**
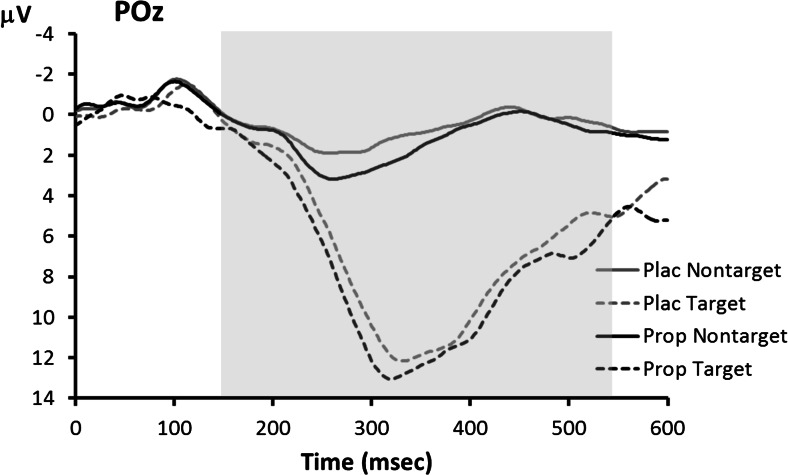
Auditory oddball task: grand average ERP waveforms associated with targets and non-targets in the placebo and propranolol condition. The *shaded area* indicates the time window used for P3 peak detection

**Fig. 6:**
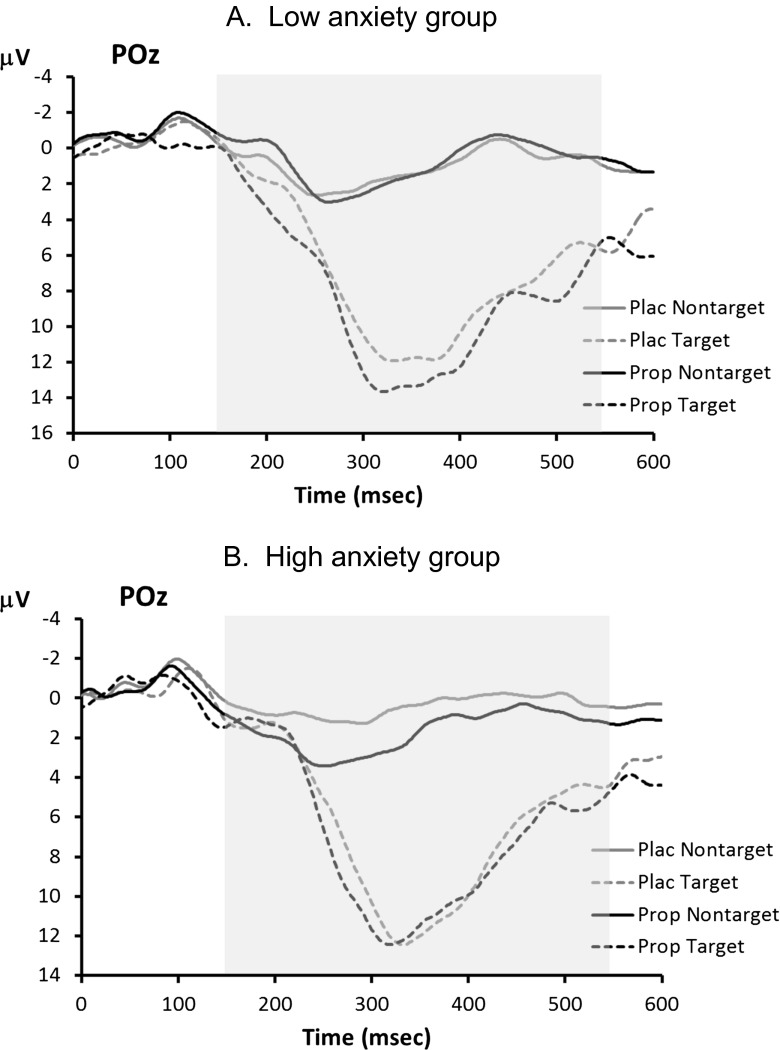
Auditory oddball task: average ERP waveforms for **a** low-anxiety participants and **b** high-anxiety participants. The *shaded area* indicates the time window used for P3 peak detection

ERP waveforms for the auditory oddball task are shown in Fig. 5. The P3 showed the typical main effect of trial type, indicating larger P3s to target stimuli than to non-target stimuli, *F*(1,12) = 588.6, *p* < 0.001, and, importantly, a main effect of treatment, *F*(1,12) = 5.3, *p* = 0.040, indicating larger P3s following propranolol treatment (placebo 8.1 μV, propranolol 9.2 μV). For the auditory task, there was no significant interaction between treatment and trait anxiety levels, *F*(1,12) = 0.04, *p* = 0.85 (Fig. 6).

### ERP flanker task

**Fig. 7:**
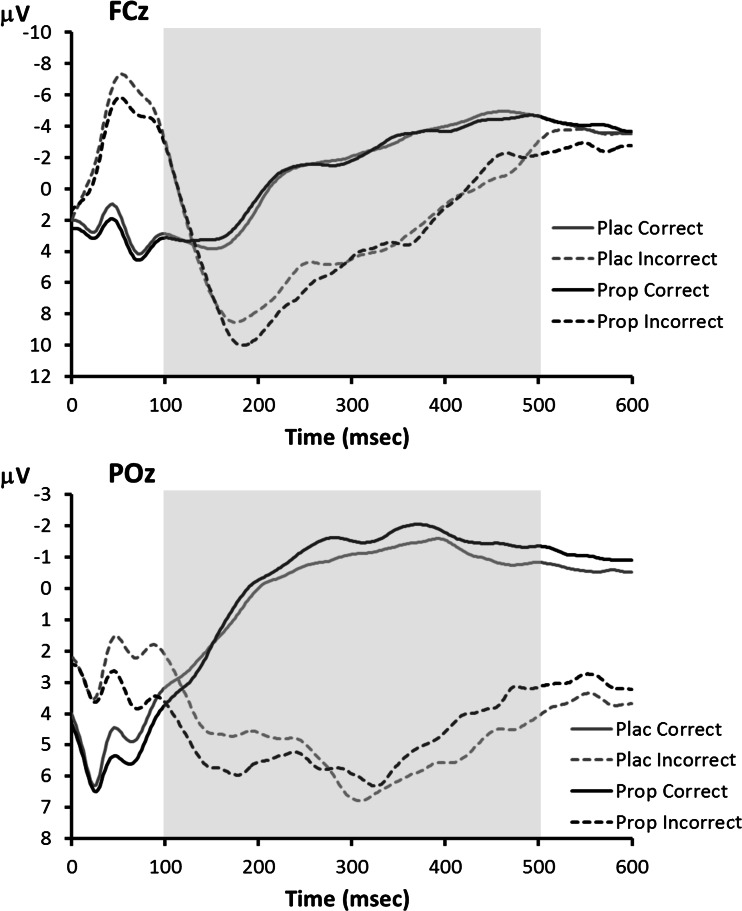
Flanker task: grand average response-locked ERP waveforms at electrodes FCz (*upper panel*) and POz (*lower panel*) associated with correct and incorrect responses in the placebo and propranolol condition. The *shaded area* indicates the time window used for Pe peak detection

ERP waveforms for the flanker task are shown in Fig. 7. There were no main effects of congruency or interactions between treatment and congruency (all *p*s > 0.4), so the waveforms shown were collapsed across this factor. In the ERN analysis, only the main effect of accuracy was significant, *F*(1,13) = 31.2, *p* < 0.001, indicating greater negativity following errors. There was no significant main effect of treatment, *F*(1,13) = 0.5, *p* = 0.50, and no significant interaction between treatment and accuracy, *F*(1,13) = 0.1, *p* = 0.71. Similar results were obtained if the ERN was defined not as the mean amplitude but as the maximum amplitude of the signal in the 0–150 ms post-response window (main effect of treatment: *F*(1,13) = 1.8, *p* = 0.20; interaction treatment × accuracy: *F*(1,13) = 1.0, *p* = 0.33).

Similarly, in the Pe analysis, only the main effect of accuracy was significant: *F*(1,13) = 22.1, *p* < 0.001, indicating a larger positivity after errors. In both analyses, there was no significant main effect of treatment (ERN: *F*(1,13) = 0.5, *p* = 0.50; Pe: *F*(1,13) = 0.1, *p* = 0.76) and no significant interactions. However, it is striking that the Pe amplitude on incorrect trials was numerically larger after propranolol treatment (placebo 10.5 μV, propranolol 11.4 μV), just like the auditory oddball P3 and the visual oddball P3 in the high-anxiety group. To examine if participants showed similar treatment effects on P3 and Pe amplitude, we correlated the treatment effects on P3 amplitude to visual and auditory targets with the treatment effect on the Pe to errors, but found no significant correlations (visual: *r* = .11, *p* = .35; auditory: *r* = −.34, *p* = .13, one-tailed), although it should be noted that the sample size was rather small for effective correlation analysis. Visual inspection (Fig. 7, lower panel) and statistical analyses suggested very similar results for the Pe at electrode POz, the electrode used for the P3 analyses.

### Behavior oddball tasks

The accuracy of responding was higher than 98 % in both tasks under all conditions and not significantly affected by treatment. Response times (RTs) on correct target trials were not significantly affected by treatment in the visual oddball task (propranolol, 311 ms; placebo, 327 ms; *t*_15_ = 1.7, *p* = .11) and in the auditory oddball task (propranolol, 316 ms; placebo, 330 ms; *t*_10_ = 0.5, *p* = .61). Given that the effect of treatment on P3 amplitude in the visual oddball task was obscured by a crossover interaction with trait anxiety level, we examined whether RTs in the visual oddball task showed a similar interaction effect. The treatment × trait anxiety level interaction was indeed significant, *F*(1,14) = 4.8, *p* = 0.045. Correct RTs were not significantly affected by propranolol in the low-anxiety group (placebo, 303 ms; propranolol, 306 ms; *t*_7_ = 0.3, *p* = .81). In contrast, correct RTs were significantly shortened by propranolol in the high-anxiety group (placebo, 351 ms; propranolol, 316 ms; *t*_7_ = 2.6, *p* = .04). This pattern more or less mirrors that for P3 amplitude and indicates that larger P3 amplitudes tended to be associated with faster RTs, consistent with previous research (e.g., Li et al. 2009). A similar modulation by trait anxiety level was not found for RTs in the auditory oddball task and flanker task, nor in other behavioral analyses, indicating a unique correspondence between P3 and RT results in the visual oddball task.

### Behavior flanker task

Trials with RTs > 1000 ms were considered outliers and discarded from analyses (0.4 % of the trials in the placebo condition and 0.2 % of the trials in the propranolol condition were discarded). RTs on correct trials were slower on incongruent trials (362 ms) than on congruent trials (338 ms), *F*(1,13) = 45.7, *p* < .001, a typical flanker interference effect. There was no significant main effect of treatment (propranolol, 347 ms; placebo, 353 ms), *F*(1,13) = 0.2, *p* = .66, and no two-way interaction, *p* = .55. Error rates showed a similar pattern: Participants made more errors on incongruent trials (18.7 %) than on congruent trials (9.7 %), *F*(1,13) = 130.1, *p* < .001. There was no significant main effect of treatment (propranolol, 13.9 %; placebo, 14.5 %), *F*(1,13) = 0.2, *p* = .66, and no two-way interaction, *p* = 0.84.

In a subsequent analysis, we computed post-error slowing by comparing correct RTs following incongruent error trials with those following incongruent correct trials. Participants showed significant post-error slowing (369 vs. 346 ms), *F*(1,13) = 12.5, *p* = .004. Post-error slowing did not differ between the propranolol and placebo conditions (both 23 ms).

Finally, we computed congruency sequence effects on correct RT and error rates. The congruency sequence effect refers to the observation that congruency effects in conflict tasks tend to be reduced following incongruent compared to congruent trials (Duthoo et al. 2014; Egner 2007). Verguts and Notebaert (2009) have proposed that the main component of this effect reflects norepinephrine-mediated Hebbian learning, presumably through actions at β-adrenergic receptors, which play an important role in associative learning. To test this proposal, we examined the effect of propranolol on the congruency sequence effect.

**Fig. 8:**
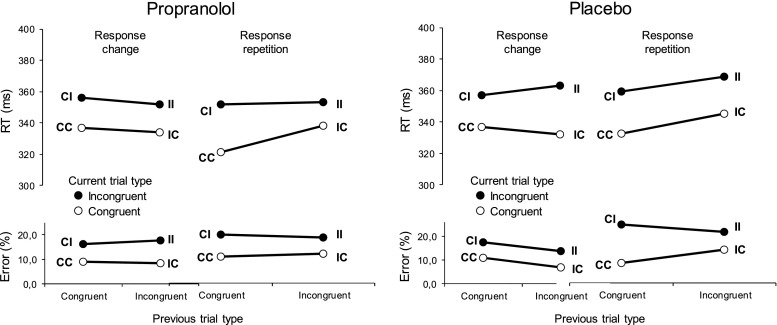
Mean response times (RTs) and error rates for each combination of current trial type and previous trial type, presented separately for response change trials (*left*) and response repetition trials (*right*). *CI*—previous trial type is congruent, current trial type is incongruent, *CC*—previous trial type is congruent, current trial type is congruent, etc. Note that a repetition of trial type (e.g., CC) can be associated with a change of response (e.g., HHHHH ≫ SSSSS), and a change of trial type (e.g., IC) can be associated with a response repetition (e.g., HHSHH ≫ SSSSS)

Trials were included in the congruency sequence analyses only if the response on the previous trial was correct and there is no outlier. Participants did not show significant congruency sequence effects in the RT data (previous × current trial type interaction, *p* = .93) and error data (*p* = .21) (Fig. 8). Replicating previous research, there was a significant three-way interaction with response type (repetition vs. alternation) in the RT data, *F*(1,13) = 7.6, *p* = . 016, and error data, *F*(1,13) = 6.3, *p* = .026, indicating a robust congruency sequence effect for response-repetition trials (i.e., trials on which the response was the same as on the previous trial, e.g., HHSHH ≫ SSSSS) but not for response-alternation trials (e.g., SSSSS ≫ SSHSS). This pattern of results suggests that the presence of congruency sequence effects reflects associative stimulus–response priming instead of conflict-driven adaptations in cognitive control (Duthoo et al. 2014). There were no reliable three-way interactions between treatment, previous and current trial type in the RT data, *F*(1,13) = 2.3, *p* = .16, and error data, *F*(1,13) = 1.4, *p* = .25, and no reliable four-way interactions in both the RT (*p* = .96) and error data (*p* = .24). The RT congruency sequence effect, defined as the difference in flanker interference effect after incongruent versus congruent trials, was 15 ms in the propranolol condition and 3 ms in the placebo condition.

## Discussion

There is a wealth of evidence suggesting an important role for the noradrenergic system in the generation of the P3 (de Taeye et al. 2014; Nieuwenhuis 2011; Nieuwenhuis et al. 2005). The primary goal of this study was to extend our knowledge of the neurochemical mechanisms underlying this relationship between noradrenergic system and P3. Previous pharmacological studies have exclusively focused on agents that affect α2-receptor signaling. Our study is the first to report β-adrenergic effects on P3 amplitude. We found that P3s to auditory stimuli in an oddball task were increased in amplitude following treatment with propranolol, a centrally acting antagonist that blocks noradrenergic signaling via post-synaptic β-receptors. Propranolol also modulated the P3 to stimuli in the visual oddball task, but the modulation went in opposite directions, depending on the participants’ trait anxiety—a correlate of baseline noradrenergic activity. In participants with lower trait anxiety, propranolol resulted in a (non-significant) decrease in P3 amplitudes. In participants with higher trait anxiety, propranolol significantly enhanced P3 amplitude. This pattern was mirrored by a similar interaction in the RT data, indicating a close correspondence between the brain and behavior. We previously found a similar interaction between propranolol and social anxiety for the late positive potential (de Rover et al. 2012),^1^ an emotion-related sustained positivity that shares some characteristics with the P3 (Hajcak et al. 2010).

It is difficult to explain these findings by referring to propranolol’s antagonist actions at post-synaptic beta receptors in noradrenergic projection areas: if activation of these receptors plays a major role in P3 generation, then it seems that blockade of these receptors should consistently decrease P3 amplitude, which is in contrast to our findings. A better explanation for our results, which also offers an account of the interaction with trait anxiety, is that propranolol decreases tonic LC activity through actions at β2-receptors in the LC (Ampatzis and Dermon 2010; Berridge and Waterhouse 2003). According to this account, the effect of propranolol on P3 amplitude depends on a subject’s baseline level of tonic LC activity, which depends both on personality characteristics and task characteristics.

**Fig. 9:**
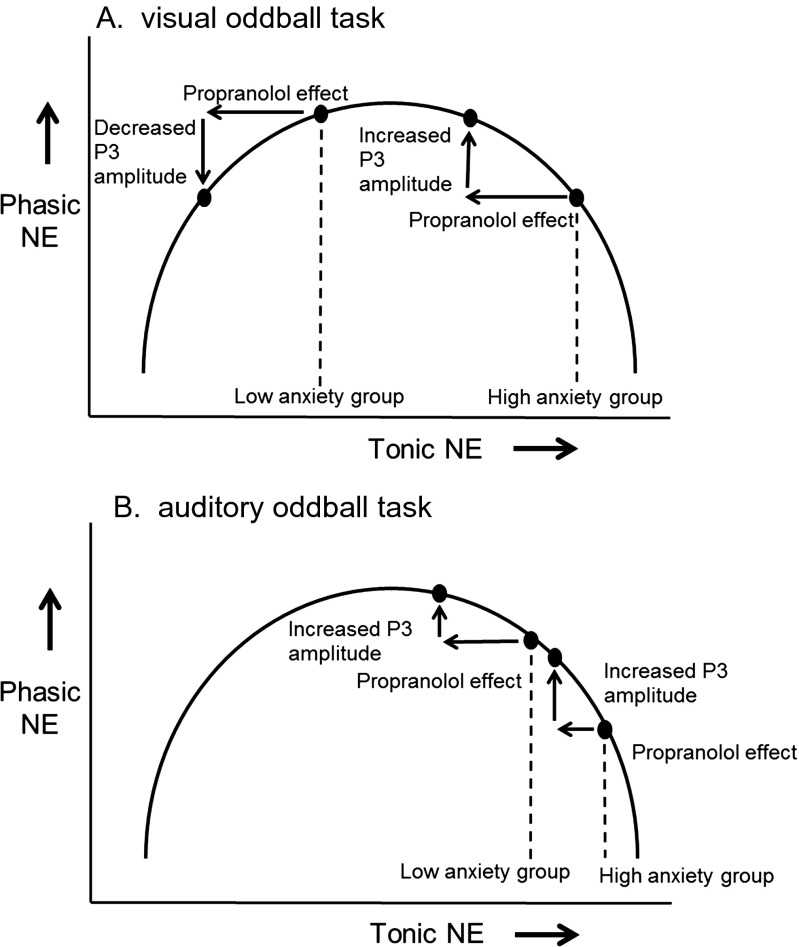
Schematic illustration of a potential mechanism underlying the observed interaction between treatment and trait anxiety, for **a** the visual oddball task and **b** the auditory oddball task. In this schematic drawing, we assumed that the effects of task and trait anxiety are underadditive

Figure 9a illustrates our account of the results in the visual oddball task. The figure shows the inverted U curve describing the relationship between tonic noradrenergic activity, which can vary from low (inattentive states) to medium (alert states) to high (stress and other high-arousal states) and phasic noradrenergic activity, which is driven by task-relevant and other motivationally significant stimuli (Aston-Jones and Cohen 2005). We assume that the high-anxiety group has a higher baseline level of noradrenergic activity than the low-anxiety group (Howells et al. 2012; Ressler and Nemeroff 2000). We also assume that in the visual oddball task, the low- and high-anxiety groups are positioned on the tonic LC axis such that propranolol shifts the low-anxiety group to a lower point on the inverted U curve describing the strength of phasic LC responses (which we assume underlies P3 generation; Nieuwenhuis et al. 2005). In contrast, propranolol shifts the high-anxiety group to a higher point on the curve. This account mirrors our explanation of the interactive effects of propranolol and social anxiety on the late positive potential (de Rover et al. 2012).

Figure 9b illustrates our account of the results in the auditory oddball task. In order to explain the results, we need to assume that participants were more aroused (i.e., had increased tonic LC activity) while performing this task, either because of the nature of the task or because the auditory oddball task was consistently performed after the visual oddball task. Unfortunately, our experiment was not designed to allow a test of this assumption: We did not record autonomic nervous system measures during task performance, and the difference in stimulation modality prevented a clean comparison of baseline EEG frequency spectra. The assumed task-related increase in arousal pushed both groups towards the right, such that propranolol shifts both groups to a higher point on the inverted U curve, thus resulting in a main effect of drug on P3 amplitude. We acknowledge that these accounts are highly speculative—our data merely show a drug × trait anxiety interaction for one task and a main effect of drug for the other task. Future research, preferably with a larger sample size and in a more heterogeneous population, should attempt to replicate our results, while including autonomic nervous system measures to empirically support assumptions about arousal.

Our pattern of results, a main effect of propranolol in the auditory task but not the visual oddball task, resembles the effects of the α2-receptor agonist clonidine on P3 amplitude in monkeys and humans. A review of this literature (Nieuwenhuis et al. 2005) shows that clonidine fairly consistently decreases P3 amplitude in auditory tasks while it does not affect P3 amplitude in visual tasks, at least at the group level. Our study raises the question whether these null effects at the group level are in fact an average of opposing drug effects for different subgroups of subjects, which differ systematically in trait anxiety or other individual difference measures. The neural mechanism may be similar to that proposed above for the effects of propranolol, given that clonidine decreases tonic LC activity through actions at α2-autoinhibition receptors in the LC. Pineda and Swick (1992) suggested that these modality-specific clonidine effects may reflect differences in the number of receptors in visual and auditory cortical areas. That is, noradrenergic fibers may preferentially innervate regions involved in auditory analysis, causing drug-induced changes in noradrenergic activity (and consequent electrophysiological effects) to be more pronounced in those areas. Although a similar account may apply to the current results, this does not address the interaction with trait anxiety in the visual oddball task. More in general, it is also unclear how our findings can be reconciled with Strange and Dolan (2007), who found that propranolol *decreased* the large BOLD response elicited by visual oddball targets, also in cortical areas known to contribute significantly to the scalp-recorded P3.

Our new evidence for a relationship between the noradrenergic system and the P3 could, in principle, inform our understanding of the role of the noradrenergic system in cognition (if we understood the cognitive process reflected in the P3) or of the cognitive process underlying the P3 (if we understood the link between noradrenergic activity and cognition). However, there is very little consensus about these topics, with prominent theories claiming a role for the P3 process in updating of memory (Donchin and Coles 1988), evidence accumulation for perceptual decision making (O’Connell et al. 2012; Verleger et al. 2005), and post-decision temporal filtering (Nieuwenhuis et al. 2005). This variety in theories is mirrored by prominent theories of noradrenergic function, which link phasic noradrenergic activity to arousal-based Hebbian learning (see Sara 2009), the accumulation of evidence for the occurrence of unexpected events (Dayan and Yu 2006), and post-decision temporal filtering (Aston-Jones and Cohen 2005). Until more consensus is reached in one of these domains, our findings mainly inform the neuromodulatory mechanism underlying P3 generation and modulation by noradrenergic agents.

Given the many parallels between the P3 and the Pe, the second aim of our study was to examine whether propranolol modulated not only the P3 but also the Pe. Although the numerical effect of propranolol on Pe amplitude was of the same magnitude and in the same direction as the propranolol effect on the auditory P3 (cf. Figs. 6 and 8), the effect on the Pe was far from significant and did not correlate across subjects with the propranolol effects on the P3. Although replication with a large sample size is warranted, our finding suggests that the generation of the Pe does not involve activation of β-receptors. A previous study that examined the effect of the α2-receptor antagonist yohimbine also found no effect on Pe amplitude (Riba et al. 2005). Future attempts to unify theories of the P3 and Pe should take into account these pharmacological findings.

Furthermore, our behavioral analyses showed that propranolol did not modulate two types of conflict/error-related adjustments. First, propranolol did not affect post-error slowing, which in recent research has been tied to a genetic marker of norepinephrine synthesis (Colzato et al. 2013). This finding is consistent with a recent study that found no effect of propranolol on post-error slowing in rats (Bari and Robbins 2013). Furthermore, propranolol did not modulate congruency sequence effects, which in our data reflect associative learning of stimulus–response pairs (Egner 2007). This finding is potentially important, because Verguts and Notebaert (2009) have hypothesized that such associative learning effects in conflict tasks reflect Hebbian learning, which is thought to occur through actions at β-adrenergic receptors (see also Brown et al. 2014). Altogether, the results in the flanker task offered no evidence for a role of the β-adrenergic system in error- and conflict-related processing.

## Footnotes

1 In this previous report (de Rover et al. 2012), the Liebowitz Social Anxiety Scale (LSAS) was used to measure anxiety. In the current study, we tested whether there was a three-way interaction between treatment, P3 (or Pe or ERN) amplitude, and LSAS score but did not find a significant interaction.
